# Utilization of Imaging to Identify a Benign Condition Mimicking Acute Appendicitis in a Child

**DOI:** 10.1177/2324709618797989

**Published:** 2018-08-31

**Authors:** Neelam Phalke, Zubin Mehta, Samrat Das

**Affiliations:** 1 University of Nevada, Reno, NV, USA; 2 University of Nevada, Las Vegas, NV, USA; 3 Department of Pediatrics, Duke University School of Medicine, Durham, NC, USA; * Current affiliation: Department of Otolaryngology, Louisiana State University School of Medicine, New Orleans, LA, USA

**Keywords:** appendicitis, omental infarct, appendagitis, inflammation

## Abstract

One of the most concerning causes of abdominal pain affecting children is acute appendicitis. However, there are benign conditions that can closely mimic appendicitis in children. In this article, we present a case of a child admitted for possible acute appendicitis and determined to have a condition known as omental infarction. The patient was managed medically and made a full recovery without surgical intervention. The aim of this case report is to review omental infarction and present a way of differentiating this disease from appendicitis, utilizing imaging, with the goal of avoiding surgical intervention. We also discuss the presentation and imaging findings of and another closely related condition—epiploic appendagitis. It is important to differentiate appendicitis from these 2 conditions as they can be often managed medically without surgical intervention.

## Introduction

Omental infarction is a benign disease process that can mimic appendicitis in children. Clinically, omental infarction often presents similarly to acute appendicitis with right lower quadrant (RLQ) tenderness, leukocytosis, elevated C-reactive protein (CRP), and anorexia. Differentiating between the two pathologies is difficult, and the majority of omental infarctions are diagnosed during surgical exploration of the abdomen for possible appendicitis revealing a normal appendix and necrotic omentum.^1,2^ Here we report the case of a child whose presentation was concerning, yet not typical, for acute appendicitis and discuss the subtle difference in clinical presentation of an omental infarct and the use of abdominal imaging to identify the disease and prevent unnecessary surgical intervention.

## Case

An overweight 9-year-old Hispanic male with no significant past medical history presented to the emergency room with 2 days of mild, persistent RLQ abdominal pain and tactile fevers for 1 day. The pain was initially intermittent, nonradiating, and mild on the pain scale, and was relieved by Pepto-Bismol. On the day of admission, the pain became constant, moderate, and was aggravated by walking, causing the patient to limp. Over the prior 2 days, he continued to attend school despite his abdominal discomfort. However, his mother noted that, while normally an avid eater, he had been avoiding food and had a reduced appetite. He complained of some nausea on the day of admission, but denied any prior nausea, emesis, or change in bowel habits.

On physical examination, the patient had stable vital signs and was in no acute distress. His abdomen was soft and nondistended. There was mild tenderness, localized to the RLQ, without peritoneal signs. He had no tenderness at McBurney’s point and negative psoas and Rovsing signs. An obturator’s test did elicit mild pain. Laboratory workup showed a leukocytosis 14 400 cells/µL with elevated CRP 109.6.

In the absence of classic signs of appendicitis with high inflammatory markers, the patient underwent computed tomography (CT) imaging to look for other intraabdominal pathology and to rule out appendicitis. Abdominal CT revealed a normal appendix and moderate inflammation of fatty structure surrounding the anterior ascending colon with mesenteric edema and pelvic ascites. These findings and a benign appendix suggested omental infarction versus epiploic appendagitis. However, CT findings of the latter would more likely show fatty inflammation of an oval-shaped paracolic mass, although definitive differentiation based on imaging may be difficult.

The patient was hospitalized and placed on intravenous fluids. Pediatric surgery was consulted, who supported a diagnosis of omental infarction. No surgical intervention was recommended. The patient was placed on complete bowel rest with serial abdominal examinations to rule out progression to overt appendicitis, given the location of the pain in the context of leukocytosis with elevated CRP. Serial abdominal examinations showed slow improvement in his discomfort over the next 24 hours with no need for pain medication. After 24 hours of inpatient stay, our patient was able to walk and even jump without significant pain. Abdominal examination showed decreased tenderness of the RLQ compared with admission and no peritoneal signs. The patient was started on a clear liquid diet and advanced to regular diet, which he tolerated without any nausea or vomiting. He was discharged home with the recommendation to take over-the-counter ibuprofen for pain control and to return if symptoms worsened.

## Discussion

The omentum is a fold of peritoneum containing vasculature, lymphatics, and fat arising from the intestinal mesentery.^3^ Rotation of the stomach during embryonic development leads to division of the omentum into a greater and lesser sac. It serves a protective function for the intestinal tract in cases of infection or injury such as a bowel wall perforation. Inflammation of the bowel causes adhesion to the omentum, thereby containing the disease process and preventing peritoneal spread.

While omental infarction has been well described in the adult literature, only a few case reports exist describing its presentation in children.^4^ Omental infarction is an uncommon and underrecognized cause of abdominal pain in children. Obesity is a significant risk factor in the pathogenesis of omental infarction. The relative paucity of intraabdominal fat and omental mass, especially in early childhood, is likely the explanation for lower incidence in children (currently 15% of all reported cases).^2,4^ Omental infarction is estimated to be the cause of acute abdominal pain in children 0.1% of the time.^5^

In both adults and children, infarction and necrosis of omental tissue due to torsion or thrombosis of omental vasculature leads to acute, sterile inflammation and pain.^1,6^ In the majority of cases, there is involvement of branches off the right gastro-epiplopic vessels, causing localized pain of the right lower abdomen.^1,3^ In secondary omental infarction, torsion is due to trauma, surgical intervention, or abdominal pathology such as the presence of a cyst or tumor.^6^

Risk factors for primary omental infarction in children include being of male gender and overweight, body mass index >85th percentile. Since obesity seems to be the most important risk factor for the development of omental infarction, the increasing rates of childhood obesity may explain the increasing prevalence of omental infarction in the recent literature.^2,5,7^ This is related to the hypothesis that an increase in fat of the redundant omentum may predispose it to twisting. Alternatively, there may be a relative ischemia of the extra fatty tissue leading to thrombosis.^1,2,4^

A diagnosis of omental infarction requires a high index of suspicion when a pediatric patient presents with atypical findings for acute appendicitis. In the case of our patient, his abdominal pain was not as severe as seen in appendicitis, reaching a maximum level of moderate pain on the pain scale, and the progression of his pain was very slow over the 2 days prior to presentation. His pain was constant and localized to the RLQ from its onset. On physical examination, he lacked the classic signs of appendicitis. This led to a consideration of other possible causes, using imaging with the aim of preventing a surgical procedure. In this case, we were able to identify a normal appendix on CT imaging as well as the inflammation surrounding the ascending colon. This led to the diagnosis of omental infarction, a benign and self-limited condition, which allowed the patient to be managed without surgical intervention.

The disease process is self-limited and management is conservative with bowel rest, pain control, and intravenous hydration. Most patients will improve progressively over the course of days.^4^ Failure to improve or worsening of symptoms is an indication for surgical resection. Primary surgical intervention of the infarcted tissue can speed recovery and decrease the risk of abscess formation, but is not necessary.^1,4^

Differentiation of omental infarction from acute appendicitis is difficult clinically, and imaging can be key in establishing its diagnosis. Children present with acute-onset lower abdominal pain, which progressively worsens but remains localized. The pain is right-sided in 80% of cases. Children may also have leukocytosis, elevated CRP, and anorexia.^1-4,6^ Compared with classical appendicitis, children with omental infarction present without fevers, gastrointestinal symptoms like nausea and vomiting, or pain originating periumbically.^4^

CT is highly sensitive (90%) and is considered the gold standard for the diagnosis of omental infarction.^2,8^ It will show a well-circumscribed, inflammatory mass located between the anterior abdominal wall and right colon with a normal appendix.^6,9^ Size of the mass varies depending on the patient’s weight. Similar to other inflammatory pathologies of the abdomen, fat stranding is present. However, compared with appendicitis or diverticulitis, there is greater fat stranding relative to bowel wall thickening since the pathology originates within the fatty omentum and the bowel wall is often spared.^6,10^ The inflammatory mass may adhere to the parietal peritoneum and a free effusion may be seen.^10,11^

In the case of children, CT imaging is not preferred due to ionizing radiation exposure, and abdominal ultrasound becomes our best tool.^12^ Abdominal ultrasound is only reported to be 60% to 80% sensitive, but is highly specific for the diagnosis of omental infarction, and it does not require radiation exposure.^2,8,13^ Furthermore, ultrasound can be used to follow the patient for infarct resolution in cases of conservative management. Ultrasonography would show an oval-shaped, noncompressible, hyperechoic mass within the omentum at the point of maximal tenderness.^2,10,13,14^ The mass may adhere to the abdominal wall depending on severity of inflammation, and free peritoneal effusion may be present.^10,11^ Visualization of a normal appendix on ultrasound can exclude appendicitis immediately.^2^ In cases where the ultrasound shows a possible omental infarction, but an appendix cannot be identified, low-dose CT imaging can be used to confirm the presence of a normal appendix. Alternatively, in one study, when the appendix was not identified on ultrasound, patients were taken for diagnostic laparoscopy in order to avoid CT ionizing radiation.^2^

Another mimicker of acute appendicitis in children worth mentioning is epiploic appendagitis, a similar benign process due to infarction of an epiploic appendage. These are 0.5- to 5-cm long, fat-filled, peritoneal sacs off the colonic wall thought to have a protective function for the colon.^15^ Epiploic appendagitis can occur primarily due to torsion of the appendage or spontaneous thrombosis of a vein or, secondarily, due to a localized inflammatory process such as appendicitis or diverticulitis.^15-17^ Patients present with a subacute lower abdominal pain, left-sided in 60% to 80% of cases, without nausea or diarrhea.^17,18^ White blood cell count and CRP are usually normal, though may be mildly elevated.^15^ Due to its more common left-sided presentation, epiploic appendagitis is more often mistaken to be diverticulitis than appendicitis.^17,19^

Imaging results are very similar to those seen in omental infarction. Diagnostic laparoscopy may be the only definitive way to differentiate the pathology, but is not necessary for management of the disease. Ultrasound would reveal an oval-shaped, noncompressible, hyperechoic mass.^16^ CT imaging will also show the ovoid, paracolic mass with a fatty core surrounded by fat stranding. Adjacent bowel wall inflammation is not usually present.^19-21^ The mass is smaller in size, given the size of an epiploic appendage compared with the omentum, and abuts the colon.^6,19^ Epiploic appendagitis is also managed conservatively with nonsteroidal anti-inflammatory drugs for pain control. Surgical intervention is avoided unless conservative management fails or a complication such as obstruction or abscess develops.^15^

## Conclusion

Omental infarction is an uncommon cause of acute abdominal pain in children, and its presentation can mimic the more prevalent and serious condition of acute appendicitis. Earlier diagnosis of this benign disease using a high index of suspicion and proper imaging can prevent a potentially unnecessary surgical procedure in children. It should be suspected in overweight children with localized RLQ abdominal pain in the absence of other findings consistent with typical appendicitis.
